# A Case Report of Pediatric Patient with Tuberous Sclerosis and Radiologically Isolated Syndrome

**DOI:** 10.3390/jcm12093284

**Published:** 2023-05-05

**Authors:** Giorgia Sforza, Gabriele Monte, Alessandra Voci, Lorenzo Figà Talamanca, Laura Papetti, Michela Ada Noris Ferilli, Martina Proietti Checchi, Massimiliano Valeriani, Romina Moavero

**Affiliations:** 1 Developmental Neurology Unit, Bambino Gesù Children’s Hospital IRCCS, 00165 Rome, Italy; giorgia.sforza@opbg.net (G.S.);; 2 Imaging Department, Bambino Gesù Children’s Hospital IRCCS, 00165 Rome, Italy; 3 Center for Sensory-Motor Interaction, Denmark Neurology Unit, Aalborg University, 922 Aalborg, Denmark; 4 Child Neurology and Psychiatry Unit, Systems Medicine Department, Tor Vergata University of Rome, 00133 Rome, Italy

**Keywords:** tuberous sclerosis complex, mTOR, multiple sclerosis, comorbidity

## Abstract

Introduction: Tuberous sclerosis complex (TSC) is an autosomal dominant neurocutaneous disease with central nervous system (CNS) involvement. Multiple sclerosis (MS) is a chronic inflammatory demyelinating disease of the CNS characterized by symptomatic episodes that occur months or years apart and affect different anatomic locations. In the absence of symptomatic episodes, radiologically isolated syndrome (RIS) could be diagnosed. Here, we report the case of a 10-year-old boy followed-up for TSC and diagnosed with RIS after a routine neuroimaging assessment. Case description: The patient was diagnosed with TSC after seizure onset at the age of 4 years. The follow-up magnetic resonance imaging (MRI) showed multiple asymptomatic demyelinating lesions. Brain and spinal cord MRI was performed after 2 months and showed additional lesions in the right frontal white matter and left cerebral peduncle, the latter with contrast enhancement. Therefore, he received a diagnosis of RIS. Visual evoked potentials were normal. Cerebrospinal fluid examination showed oligoclonal bands. The search for AQP4-IgG and MOG-IgG antibodies was negative. He was treated with interferon beta-1a. Six months later, follow-up MRI revealed no new demyelinating lesions and resolution of contrast enhancement. Conclusion: To the best of our knowledge, this is the third reported patient presenting a co-occurrence of TSC and demyelinating disease. Although we cannot state if the described comorbidity is casual or not, some clinical and preclinical data suggest that the mTOR complex might be the link between TSC and demyelinating disease.

## 1. Introduction

Tuberous sclerosis complex (TSC) is an autosomal dominant neurocutaneous disease, affecting about 1 in 6000 newborns and caused by a mutation in one of the two genes *TSC1* and *TSC2* [1], respectively encoding for hamartin and tuberin. These proteins are implicated in the regulation of cell proliferation and differentiation, forming a complex that activates guanosine triphosphatase (GTPase) while keeping the Ras homolog enriched in the brain (RHEB) inactive with the aim of inhibiting the mammalian target of rapamycin (mTOR) [2]. This complex is a crucial controller of protein and lipid biosynthesis and cell-cycle progression associated with the growth factor, and plays a key role in neurodevelopment [3,4]. Under ordinary circumstances, hamartin and tuberin are activated via biosynthetic processes mediated by the mTOR complex 1 (mTORC1), which includes mTOR, raptor (protein associated with mTOR regulation), mLST8, and PRAS40 (proline-rich Akt substrate 40) [5]. Therefore, *TSC1* or *TSC2* mutations give rise to hyperactivation of the mTOR pathway, resulting in a downstream kinase signaling cascade that can lead to abnormalities in numerous cellular processes, including cell cycle progression, transcription, translation, and metabolic control [5]. mTORC1 signaling has been demonstrated to be early activated during brain development, and pathological neural networks are therefore already present in the prenatal period. This early overactivation of the mTOR pathway is also responsible for abnormal neuronal migration and microstructural changes, responsible for both epileptogenicity and abnormal neurodevelopment [6]. It has been found to be linked to epilepsy and autism spectrum disorders (ASD), thus representing the neurobiological substrate of almost all TSC-related manifestations [4,7]. TSC is characterized by the development of hamartomatous lesions in various organs and systems, including brain, skin, kidneys, heart, and eyes [8]. The central nervous system (CNS) is almost inevitably involved. One of the hallmarks of TSC are cortical malformations referred to as cortical tubers. Other TSC-related brain lesions include subependymal nodules, subependymal giant cell astrocytomas and radial migration lines [9]. Furthermore, more advanced magnetic resonance imaging (MRI) techniques allowed for the detection of several and diffuse alterations in the so-called normal-appearing white matter, probably reflecting focal migration abnormalities [10,11]. Finally, diffusion tensor imaging studies unraveled widespread white matter alterations reflecting dysconnectivity [12,13]. From a clinical point of view, up to 85% of patients with TSC present with epilepsy, and more than 90% of patients present at least one neuropsychiatric disorder (including learning disability, ASD, attention deficit hyperactivity disorder, mood and/or anxiety disorders and academic difficulties) throughout their life [9]. Although the underlying neuropathological substrate of these neuropsychiatric conditions are not known, it has been demonstrated in different studies that diffuse alterations in white matter seem to be associated with higher rates of neuropsychiatric comorbidities [14,15,16].

Multiple sclerosis (MS) is a chronic inflammatory demyelinating disease of the CNS. Disease onset under 18 years of age (i.e., pediatric-onset MS, POMS) occurs in 3–10% of all MS patients [17]. The hallmark of MS is symptomatic episodes that occur months or years apart and affect different anatomic locations. In the absence of symptomatic episodes, radiologically isolated syndrome (RIS) could be diagnosed if MRI shows demyelinating lesions highly suggestive of MS. The definition of RIS was first proposed in 2009 [18] and was presented for the pediatric population in 2017 by Makhani et al., utilizing the 2010 McDonald criteria definition of dissemination in space (DIS) [19]. After the 2017 revisions of the McDonald criteria, the RIS diagnostic criteria require a demonstration of the revised DIS: one or more T2-hyperintense lesions in at least two of four MS-typical regions of the CNS (i.e., periventricular, cortical or juxtacortical, infratentorial, and spinal cord) [20,21]. RIS is excluded if there is clinical evidence of neurologic dysfunction suggestive of MS based on historical symptoms and/or objective signs. Approximately one third of individuals with RIS are diagnosed with MS within 5 years of presentation in both adults and children [19,22]. RIS is rare in the general population, particularly in children, with a neuroimaging study reporting a prevalence of 0.03% in a general pediatric population [23]. The risk factors of developing MS are younger age, male sex, higher cerebral lesion load, asymptomatic infratentorial or spinal cord lesions, gadolinium-enhancing lesions, the presence of oligoclonal bands in the cerebrospinal fluid (CSF) and abnormal visual evoked potentials (VEPs) [20]. Spinal cord lesion and oligoclonal bands in the CSF are another risk factor confirmed at pediatric age [19]. There is no clear indication for the use of disease-modifying treatment (DMT) for patients with RIS at present. However, it is important to consider that POMS, compared to adult-onset MS, exhibits a higher relapse rate, a faster accumulation of MRI lesions early in the disease course, and an increased risk of long-term disability [24,25].

The etiology of MS is still largely unknown, but it is likely that it begins as an immune-mediated inflammatory disease sustained by autoreactive lymphocytes in a genetically susceptible individual, possibly triggered by environmental factors [26]. To the best of our knowledge, the pathogenesis of TSC and demyelinating diseases seems to be distinct. Here, we report the case of a 10-year-old boy followed-up for TSC and diagnosed with RIS after a routine neuroimaging assessment.

## 2. Case Description

**Figure 1:**
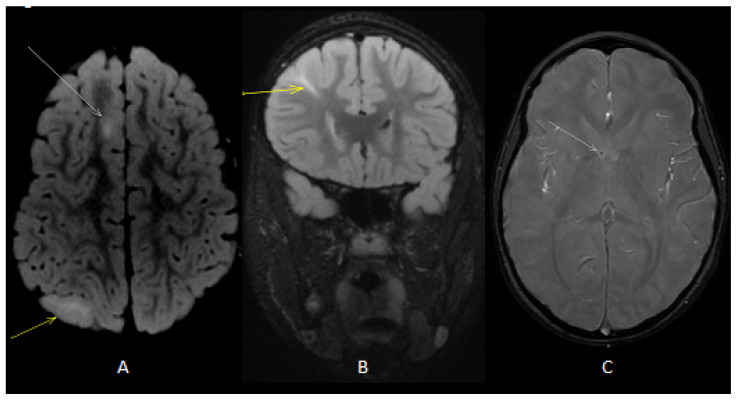
Axial (**A**) T2 fluid-attenuated inversion recovery (FLAIR) image showed cortical (white arrow) and subcortical (yellow arrow) tubers. Coronal (**B**) T2 FLAIRimage showed radial band (yellow arrow). Axial T2 gradient echo (GE) image (**C**) showed subependymal calcific nodules along lateral ventricles (white arrow).

**Figure 2:**
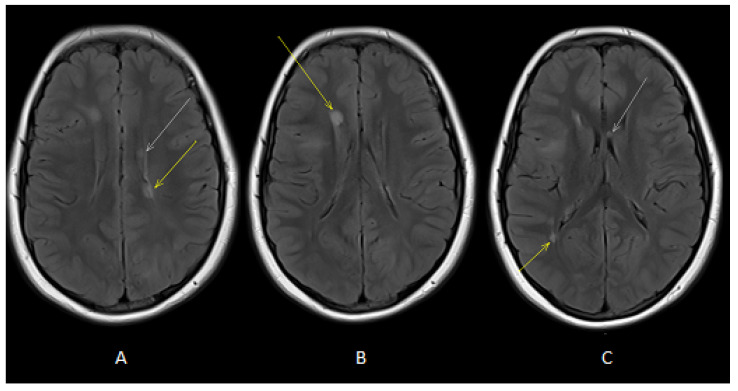
Axial T2-FLAIR images (**A**,**B**) showed oval-shaped lesions in deep periventricular white matter of both cerebral hemispheres (white and yellow arrows). Axial T2-FLAIR image (**C**) showed oval-shaped lesions in deep periventricular white matter (yellow arrow) and in the corpus callosum (white arrow).

**Figure 3:**
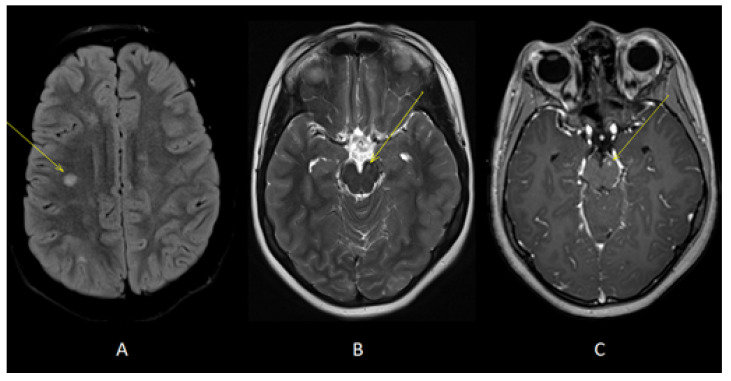
Axial T2-FLAIR (**A**) and T2 (**B**) images showed additional lesions in right deep frontal white matter (yellow arrow, **A**) and left cerebral peduncle (yellow arrow, **B**). Axial T1 images acquired after gadolinium administration (**C**) showed enhancement of the lesion located in left cerebral peduncle (yellow arrow).

Our patient is a male born at term from healthy, non-consanguineous parents. The first motor developmental milestones were acquired on time, but he presented a language delay. Later on, socio-relational difficulties and hyperkinesia were evident, and he was diagnosed with ASD and attention-deficit hyperactivity disorder (ADHD). At the age of 4 years, he began to present tonic seizures characterized by loss of awareness and dystonic posture of the upper extremities. He therefore underwent different examinations that led to a definite diagnosis of TSC [27]. Brain MRI revealed multiple cortical and subcortical tubers in both cerebral hemispheres associated with radial migration lines and subependymal calcific nodules along lateral ventricles and at the right foramen of Monro (Figure 1). Systemic investigations were carried out, allowing for the detection of cardiac rhabdomyomas and multiple bilateral renal angiomyolipomas. Molecular analysis revealed a de novo mutation in the *TSC1* gene (c.181del p. (Leu61 *)). Periodical follow-up according to international recommendations was planned. Treatment with carbamazepine was started, with good response and no more seizures, so that, at the age of 9, antiseizure treatment was withdrawn. However, a few weeks after carbamazepine was tapered off, seizures appeared again, characterized by brief psychomotor arrest and he restarted treatment with benefit. At the age of 10 years, the annual brain MRI revealed stable TSC-related brain lesions but also the presence of multiple oval-shaped lesions in the deep periventricular white matter of both cerebral hemispheres and in the corpus callosum (Figure 2) without contrast enhancement (CE). An acquired inflammatory demyelinating disease of CNS was suspected. A follow-up brain and spinal cord MRI was performed after 2 months and showed additional lesions in the right frontal white matter and in left cerebral peduncle, with the latter showing CE (Figure 3). Neurological examination was unchanged, with a lack of both neurological signs and subjective symptoms. Although we know that epileptic seizures can be part of the clinical pattern of MS, the temporal relationship with the carbamazepine withdrawal led us to hypothesize that the seizures were still linked to TSC, although nothing could be determined with certainty. Furthermore, we are fully aware that the presence of a neurodevelopmental disorder could interfere with the patient’s ability to refer symptoms such as sensory disturbances; however, the lack of any complaint from him and his family led us to consider him as asymptomatic. Lumbar puncture was performed, and CSF chemical analysis showed normal results. CSF cultures and polymerase chain reaction (PCR) showed no evidence of infection. Viral capsid antigen (VCA)-IgG and Epstein–Barr virus (EBV) nuclear antigen (EBNA)-IgG was found, suggesting a previous EBV infection. Oligoclonal bands were found. VEPs were normal. The search for anti-aquaporin-4 (AQP4) and anti-myelin oligodendrocyte glycoprotein (MOG) antibodies in serum was negative. Therefore, he received an RIS diagnosis according to 2017 McDonald’s criteria, as T2-hyperintense lesions were present in two of the four MS-typical regions of the CNS (i.e., periventricular and infratentorial) in the absence of clinical evidence suggestive of MS [20,21]. Due to the presence of a CE lesion, a high dose of intravenous methylprednisolone (20 mg/kg/day) was administered for three days. Considering the presence of several risk factors (i.e., young age, asymptomatic infratentorial lesion, gadolinium-enhancing lesions, presence of oligoclonal bands) for the development of MS and the presence of new lesions at follow-up MRI, we decided to start interferon (IF) beta-1a 44 mcg subcutaneously three times weekly. Six months later, follow-up MRI revealed no new demyelinating lesions and resolution of CE. TSC-related lesions were stable. The patient was still asymptomatic, but he complained of some adverse effects of interferon, including fever, pain and psychomotor agitation. Therefore, we decided to switch to interferon beta-1a 30 µg intramuscularly once a week with improved tolerability.

## 3. Discussion

To the best of our knowledge, this is the third reported patient presenting a co-occurrence of TSC and demyelinating disease, since two patients with concurrent TSC and MS have recently been described [28]. The main characteristics and diagnostic tests of these three patients are summarized in Table 1.

It is difficult to find a possible pathogenic link explaining this comorbidity, and we cannot exclude the possibility that this is a mere coincidence. Indeed, the two diseases have different pathophysiological mechanisms, although the mTOR complex might be a potential link. MS is a heterogeneous disease with unknown etiology; however, the most widely accepted theory is that MS begins as an immune-mediated inflammatory disease caused by autoreactive lymphocytes. Thereafter, the disease is dominated by microglial activation and chronic neurodegeneration [16]. The hamartin–tuberin complex inhibits cell signaling mediated by the mTOR [29]; thus, mTOR plays a key role in regulating protein translation, responding to hypoxia and cell-cycle progression. For refractory epilepsy, subependymal giant cell astrocytomas (SEGAs) and renal angiomyolipomas, there is a target therapy with the mTOR inhibitor, called everolimus [30]. The deregulated mTOR pathway has a clear pathogenic mechanism in TSC, but there is also evidence of a role in demyelinating disease, particularly in MS pathogenesis [31]. The signaling pathways regulated by mTOR are involved in autophagy, inflammasome activation, innate and adaptive immune responses, axonal and neuronal degeneration, and its dysregulation is reported in cancer, metabolic disorders, neurological and inflammatory disorders [32]. Increased mTOR activation has been shown in MS patients, but it is known that mTOR inhibition could have both pro- and anti-inflammatory effects [33]. Interestingly, in vivo models where these opposing actions take effect simultaneously demonstrate that mTOR inhibitors ameliorate experimental autoimmune encephalomyelitis (EAE) (i.e., the animal model of MS). This could be related to the reduction in T-effector development and function-limiting-related autoimmunity and to the possible protective effect on neurons, astrocytes and oligodendrocytes connected to autophagy induction [34,35]. Inhibiting mTOR activity could result in myelin recovery and limit the progression of MS [35]. However, reports of mTOR-inhibitor effects in MS patients are limited to small clinical trials and cohort studies. In conclusion, although we cannot state if the described comorbidity is casual or not, some clinical and preclinical data suggest that the mTOR complex might be the link between TSC and demyelinating disease.

## Figures and Tables

**Table 1:** Characteristics and diagnostic tests of the three patients presenting a co-occurrence of TSC and demyelinating disease.

	Patient 1 (Sforza G. et al.)	Patient 2 (Quigley S. et al. [28])	Patient 3 (Quigley S. et al. [28])
Age (years)	10	18	16
Sex	Male	Female	Male
TSC			
TSC gene	*TSC1*	*TSC1*	*TSC2*
Epilepsy	Yes	Yes	No
Neuropsychiatric disorder	Yes	No	Yes
Demyelinating disease			
Clinical relapse	No	Yes	Yes
Optic neuritis	No	Yes	No
Brain MRI	DL	DL	DL
Spinal cord MRI	Normal	DL	DL
CSF chemical analysis	Normal	NA	Normal
OCB	Positive	Positive	Positive
VEPs	Normal	NA	NA
Anti-AQP4 Abs	Negative	Negative	Negative
Anti-MOG Abs	Negative	Negative	Negative

Abs: antibodies; AQP4: aquaporin-4; CSF: cerebrospinal fluid; DL: demyelinating lesions; MOG: myelin oligodendrocyte glycoprotein; MRI: magnetic resonance imaging; NA: not available; OCB: oligoclonal bands; TSC: tuberous sclerosis complex; VEPs: visual evoked potentials.

## Data Availability

The data presented in this study are available on request from the corresponding author.
